# The Use of Beetroot Juice as an Impregnating Solution to Change Volatile Compounds, Physical Properties and Influence the Kinetics of the Celery Drying Process

**DOI:** 10.3390/molecules29174050

**Published:** 2024-08-27

**Authors:** Magdalena Kręcisz, Marta Klemens, Aleks Latański, Bogdan Stępień

**Affiliations:** 1 Institute of Agricultural Engineering, Wroclaw University of Environmental and Life Sciences, Chełmońskiego Street 37a, 51-630 Wrocław, Poland; 120609@student.upwr.edu.pl (A.L.); bogdan.stepien@upwr.edu.pl (B.S.); 2 Department of Food Chemistry and Biocatalysis, Wrocław University of Environmental and Life Sciences, Norwida 25, 50-375 Wrocław, Poland; marta.klemens@upwr.edu.pl

**Keywords:** kinetics, vacuum impregnation, celery, beetroot, drying, VOCs

## Abstract

The effect of different methods of drying celery root enriched with beet juice by vacuum impregnation (VI) was studied. The process of convection drying, vacuum drying and freeze drying was carried out. Compared to dried indigenous celery, dry impregnated tissue was characterized by lower values of dry matter, L* and b* color parameters, as well as higher values of water activity, density and a* color parameter. In addition, VI reduced the drying time. Forty Volatile Organic Compounds (VOCs) were found in celery, while fifty-one VOCs were found in the profile of celery with beetroot juice. The innovative method of vacuum impregnation made it possible to produce a new type of product with changed properties and a variable VOCs profile. The best fit of the drying process kinetics was achieved by using the logistic model. Increasing the temperature during convection drying resulted in shorter drying time, increased values of dry matter, reduced the water activity value and altered VOCs.

## 1. Introduction

The consumption of vegetables is currently highly recommended in the diet, so it is desirable to develop new high-quality dried vegetables that are attractive to consumers, in order to expand the availability of products and diversify their market [1]. For this purpose, it is necessary to optimize drying conditions and use pretreatment to obtain specific properties related to color, water content, density, texture and volatile compounds, etc. [2,3]. Modern society is increasingly paying attention to a healthy lifestyle, placing importance on choosing conscious and functional food products [4]. Awareness of the effects of diet on overall health makes consumers care not only about taste but also about the nutritional value of the products they eat. In this context, the popularity of celery, which is rich in nutrients and has numerous health-promoting properties, is gaining ground [5].

Celery root is a vegetable derived from the celery family (*Apiaceae*) and is widely used around the world [6,7]. There are many ways to consume celery root. Celery can be boiled, stewed, roasted or eaten raw. The most common color of celery root is white, yellow or green depending on the variety. It is low in calories, which makes it ideal for people who want to maintain or lose weight. Celery root is one of the few vegetables with a high fiber content, which aids in digestion and maintaining a healthy digestive system. It is rich in many nutrients (potassium—320 mg/100 g, vitamin C—6.2/8.0 mg/100 g, folate—12 mg/100 g, total sugars—2.25% and vitamin—K 100 mg/100 g) [7]. All parts of celery have medicinal properties, i.e., anti-inflammatory, antibacterial and able to lower serum lipids and blood glucose levels. In addition, celery prevents cardiovascular diseases and strengthens the heart [6,8]. In addition, due to its numerous volatile compounds, celery has a peculiar aroma and taste [9].

The drying of vegetables and fruits is one of the prevailing methods for the manufacture and development of novel products, as it extends their shelf life and does not significantly degrade the quality of products with a high moisture content [10,11]. Extended drying time can effect undesirable physicochemical changes and low energy efficiency in food products. For this reason, innovative and cutting-edge approaches to the drying process and pretreatment prior to drying are key, which can improve the quality of biological materials, reduce drying time and increase the overall efficiency of the drying process [10]. Pretreatment plays an important role in accelerating the drying rate, oxidation, permeability and enzyme inactivation of many vegetables and fruits [12].

Vacuum impregnation (VI) is a process that allows the development of new foods by changing their composition and affects the final characteristics of the product. During the vacuum impregnation process, there is an internal exchange of gas and liquid present in the pores with an external impregnating solution [3]. This occurs due to a mechanically induced pressure difference. The VI process consists of two stages: in the first stage the material is subjected to reduced pressure and in the second stage to atmospheric pressure. As a result of these phenomena, the intracellular capillaries are filled with the impregnating solution [13]. The process of vacuum impregnation can contribute to the modification of the properties of vegetables in terms of nutrition, sensory qualities, physicochemical values of color and water content, as well as changes in texture, antitussive and bioactive properties [13,14,15,16]. Therefore, the process is widely used in the functional food industry and can affect final product properties and drying characteristics [3].

Research available in the literature on celery is mainly related to its breeding, cultivation and chemical composition. Celery is most commonly used in daily vegetable consumption. Despite numerous publications on the health-promoting properties of celery, there is a lack of other celery food products on the market [6]. Therefore, the purpose of this study was to investigate the effects of different drying techniques and parameters of celery root and vacuum-impregnated celery root on selected physical properties, volatile compounds and kinetics of the drying process.

## 2. Results and Discussion

The results and discussion section presents the results of the study of celery (C) and celery after the vacuum impregnation process (VI) using beetroot juice (CB). In the presented study, three drying methods were tested: freeze drying (FD), vacuum drying (VD) and convection drying at three temperatures of 50 °C (CD50), 60 °C (CD60) and 70 °C (CD70).

### 2.1. Volatile Organic Compounds (VOCs) Profile

The beetroot impregnation procedure was carried out on celery, which was then subjected to different drying methods and the VOC profiles were compared with each other using the HS-SPME Arrow technique. The full VOC profiles of the samples are shown in Table 1 and Table 2. Forty VOCs were found in celery, while fifty-one VOCs were found in the profile of celery with beetroot juice.

In the case of fresh celery, the highest amount of volatile compounds were limonene (48.54%), β-pinene (13.4%), p-cymene (7.53%), β-(*E*)-ocimene (7.42%) and pentyl cyclohexa-1,3-diene (6.37%). The same compounds were repeated in fresh beetroot-impregnated celery: limonene (42.72%), β-pinene (18.16%), p-cymene (9.01%) and β-(*E*)-ocimene (8.37%).

**Figure 1:**
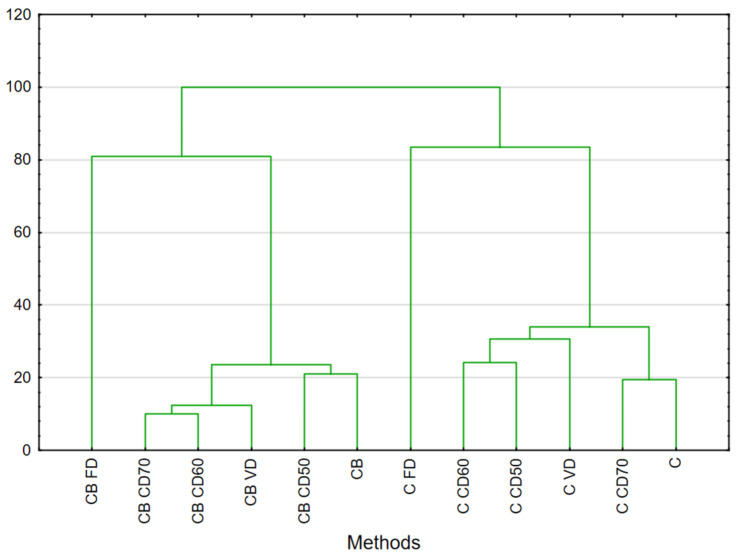
HCA result analysis of celery and celery with beetroot juice. Celery (C), celery after vacuum impregnation with beetroot juice (CB), control (F), material after freeze drying (FD), vacuum (VD) and convection drying at a temperature of 50 °C (CD50), 60 °C (CD60) and 70 °C (CD70).

Our previous studies have shown that the issue of impregnation varies depending on the plant material. In the studies on sweet potato, we observed interesting changes in the profile [16], but in this case, as with courgette and broccoli [2], there were no significant alterations. In previous cases, we used the juices of onion, kale and celery stalks to impregnate celery [15] and the effect was significant. Meanwhile, the use of beetroot juice did significantly change the volatile compound profile in comparison with fresh, pure celery, which was shown in Figure 1 presenting HCA results. The amounts of major compounds such as limonene (36.02%) and pentyl cyclohexa-1,3-diene (3.22%), decreased after impregnation. In celery after vacuum impregnation, an increase in the content of compounds such as Fenipentol (2.74), γ-Terpinene (3.71) and β-(*E*)-Ocimene (7.47) and the appearance of compounds such as (E)-Caryophyllene (0.41), trans-Geranylacetone (4.17), (2*E*)-Decenal (0.56), (5*Z*)-Octen-1-ol (15.86), Caryophyllene oxide (36.02), cis-9-Tetradecen-1-ol (7.47) and cis-Limonene oxide (3.71) were observed. Convection drying of celery at 50 °C and 60 °C had the same effect. For the main compounds, freeze-drying proved to be the worst method because limonene (9.80%), β-pinene (2.39%) or β-(*E*)-ocimene (1.95%) decreased significantly; however, compounds such as tetradecane (15.28%), fenipentol (7.28%), dodecane (31.98%) and decanal (8.54%) increased. In the case of freeze-drying, the method used has a greater impact on the dried raw material than the addition of beetroot juice.

Surprisingly, impregnation of celery with beetroot before convection drying at 50 °C brought a compound profile similar to fresh celery. This makes it possible to argue that its addition can maintain the natural flavor of celery.

The other methods for celery and celery with beetroot juice, such as vacuum drying at 60 °C or convection drying of celeriac after impregnation at 60 °C and 70 °C, were not significantly different, and the aroma profile was similar.

### 2.2. Drying Kinetics

The results of the statistical analysis and the constants and coefficients of the five proposed models used to describe the kinetics of the convection drying process of celery (C) and celery after vacuum impregnation (CB) are shown in Table 3. To match the experimental data with the selected drying models, the following statistical parameters were analyzed to determine the best model: R^2^, χ^2^, V_e_ and RMSE. It can be observed that a good fit to the experimental data was obtained for each of the analyzed models. The coefficient of determination (R^2^) of equations ranged from 0.9903 to 0.9994. The RMSE and reduced test (χ^2^) values were small, ranging from 0.0084 to 0.0370 and 0.0001 to 0.0016, respectively. The best fit to the experimental data was obtained for the logistic model, as the R^2^ was the highest, and RMSE, V_e_ and χ^2^ were the lowest. 

**Figure 2:**
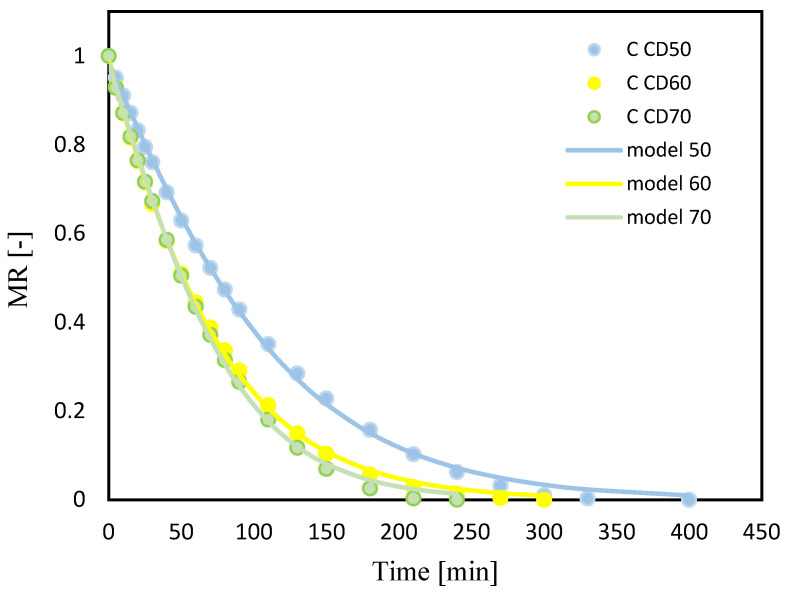
Celery (C); drying kinetics for convective drying at 50 °C (CD50), 60 °C (CD60) and 70 °C (CD70). MR—moisture ratio.

**Figure 3:**
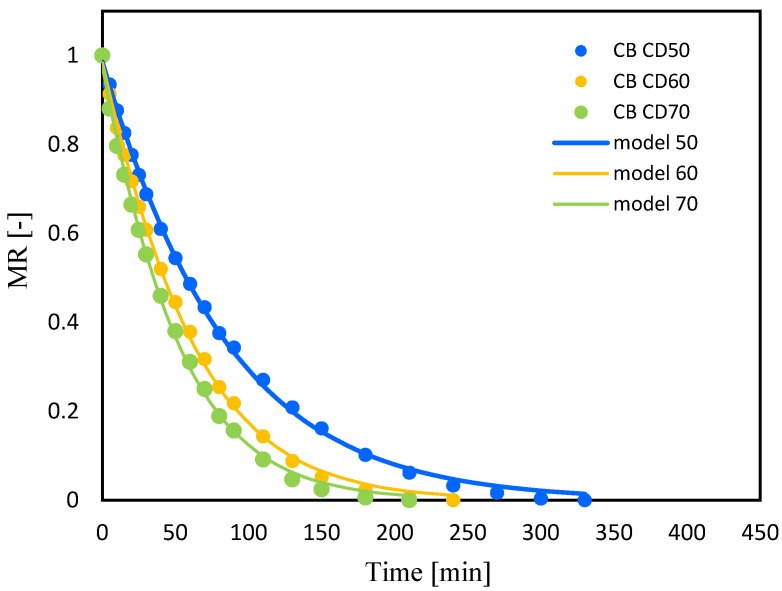
Celery after VI with beetroot juice (CB); drying kinetics for convective drying at 50 °C (CD50), 60 °C (CD60) and 70 °C (CD70). MR—moisture ratio.

The drying kinetics curves of celery root (C) and celery after vacuum impregnation with beet juice (CB) at different temperatures of the drying medium are shown in Figure 2 and Figure 3. Drying continued until a constant water content was reached. The moisture content of celery root decreased rapidly during the first 20 min. The rapid evaporation of water at the initial stage of drying may be due to the fact that the moisture in the samples at the early stage of drying was mainly on the surface of the sample, so it could evaporate quickly [17]. In subsequent stages of drying, the water loss time was extended. The water on the surface of the samples evaporated quickly, and the water inside the celery moved to the surface, which can occur through capillary movement, liquid diffusion, gas diffusion or surface diffusion [18].

Increasing the temperature of the drying medium from 50 °C to 70 °C significantly reduced the drying time for both celery without pretreatment and celery impregnated with tomato juice, by 40% and 36%, respectively. Krzykowski et al. [19], subjecting Wild Strawberry *Fragaria vesca* L. to drying, observed that increasing the convection drying temperature from 25 °C to 60 °C reduced drying time by 60%. Other authors also observed a reduction in drying time with an increase in the temperature of the convection process by subjecting jackfruit [18], bitter gourds [12] and coriander [20] to drying.

In the case of celery drying, pretreatment affected the drying time at all three temperatures during convection drying (50, 60 and 70 °C). Thus, the drying period of celery subjected to vacuum impregnation with beet juice reduced the drying time, which is consistent with the results of studies of drying figs [21] and cherry tomatoes [22]. Studying the effect of blanching on the drying process of mangoes, researchers observed a reduction in drying time from 14.46% to 19.95% depending on the drying method [23]. Similar observations were noted by other authors studying the effect of blanching on the drying time of celery [24].

**Figure 4:**
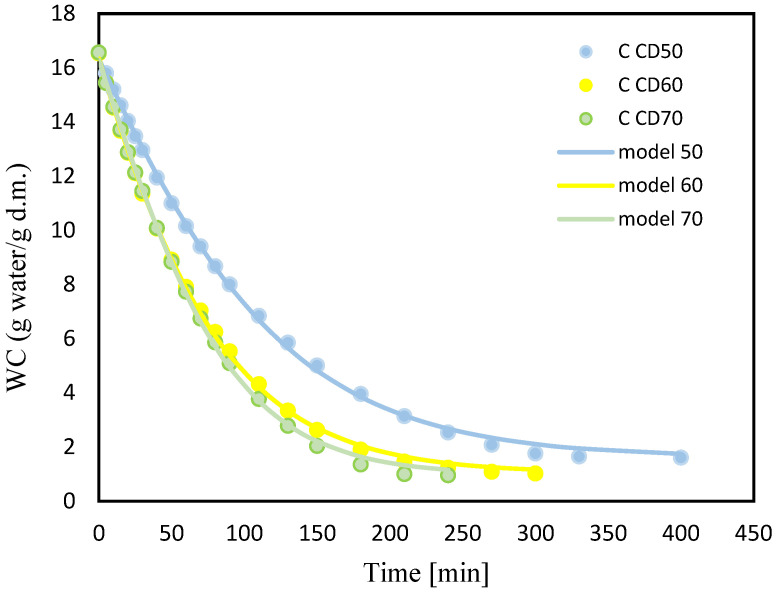
Changes in water content (WC) for celery (C) with drying time for convective drying at 50 °C (CD50), 60 °C (CD60) and 70 °C (CD70).

**Figure 5:**
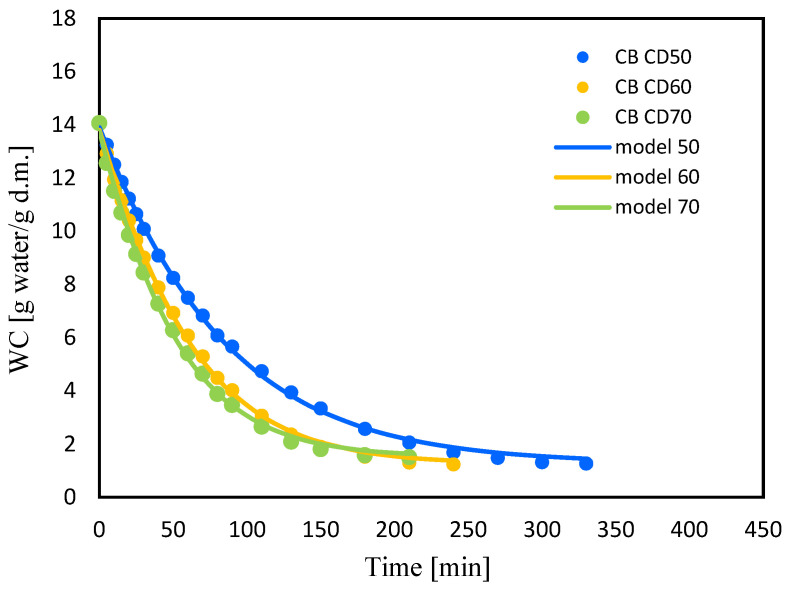
Changes in water content (WC) for celery after VI with beetroot juice (CB) with drying time for convective drying at 50 °C (CD50), 60 °C (CD60) and 70 °C (CD70).

Figure 4 and Figure 5 show changes in water content (WC) with drying time during convection drying at 50 °C (CD50), 60 °C (CD60) and 70 °C (CD70). It is clear that the moisture content of celery and celery samples after the vacuum impregnation process showed a general trend of decreasing with increasing drying time. It was noted that celery without pretreatment had a higher water content of 16.7 (g water/g dry weight). The application of the vacuum impregnation process reduced the water content of the samples after the vacuum impregnation process to 14 (g water/g dry weight). Kręcisz et al. [14] recently showed that the moisture content of zucchini decreased after applying impregnating solutions such as onion juice, kale juice and a mixture of onion and kale juices (50:50), as well as with the addition of a 3% NaCl solution. Similar observations were noted in our previous studies on dried celery [15].

### 2.3. Dry Matter (DM), Water Activity (AW) and Density (ρ_b_)

The dry weight, water activity and density of fresh celery (C), vacuum-impregnated celery (CB) and the resulting dries are shown in Table 4. For dried celery, the lowest dry mass value was observed for CB CD50 (87.69%) and the highest was for C FD (99.26%). Theoretically, the growth of microorganisms is inhibited below the level of 20% moisture [25]. However, a study presented by Singh et al. [23] shows that a moisture content of 15% in mangoes is considered safe. The use of the VI process significantly affected the dry weight of dried celery root, causing a decrease in the studied parameter from 1 to 5% depending on the drying method used. The drying method significantly affected DM. The highest values were recorded for dries obtained by the sublimation method (99.26%) and the lowest for dries obtained by the convection method at 50 °C. Increasing the drying temperature from 50 °C to 70 °C increased the dry weight from 8 to 9%, depending on the material used.

Water activity (Table 4) in dried celery root ranged from 0.110 to 0.557 (for C FD and CB CD50, respectively). Application of the vacuum impregnation process significantly increased AW in all tested samples compared to celery not subjected to VI. When stowing impregnating solutions containing more than 30 °Brix, a decrease in water activity is observed in the tested material [13]. The lowest AW values were recorded for FD for both C and CB. The lowest water activity values for freeze drying were recorded by other authors studying apples [26], Increasing the process temperature yielded dries with lower water activity.

The bulk density for fresh materials ranged from 221.32 kg/m^3^ for celery without VI and 271.43 kg/m^3^ for celery after VI. On the other hand, the density of dried materials for celery without pretreatment and for celery after vacuum impregnation ranged from 34.28 kg/m^3^ to 72.04 kg/m^3^ and from 34.48 kg/m^3^ to 94.24 kg/m^3^, respectively. The lowest density value was recorded during freeze drying for C and CB, and it can also be noted that their difference is relatively not great, but the materials after vacuum impregnation have a higher value of the studied parameter. The lowest drying density after FD was also observed by other researchers who compared drying methods for apples [26]. Convection drying at 50, 60 and 70 °C temperatures showed a significant effect of temperature on the efficiency of the process. As the temperature of the drying medium increases, a reduction in the density of the product is observed, which can be beneficial, especially in the production of food or food materials for packaging. The vacuum impregnation process contributed to an increase in the density of celery in each of the tested materials and for each of the drying methods.

### 2.4. Color

Food color and its acceptability at the time of purchase have a direct impact on the identification and ultimately on the decision of consumers. Therefore, it is important that the darkening reaction and pigment damage do not occur during food processing [27]. Table 5 shows the color characteristics of fresh and dried samples.

**Figure 6:**
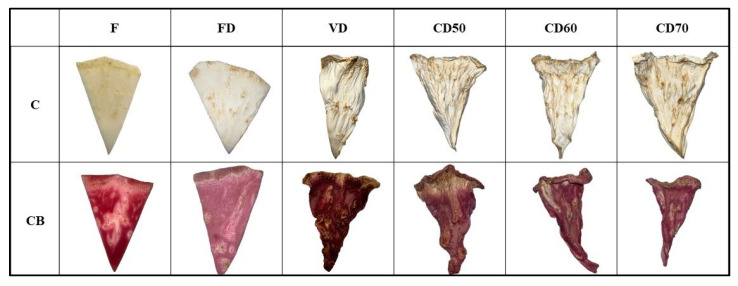
Celery (C), celery after vacuum impregnation with beetroot juice (CB), control (F), material after freeze drying (FD), vacuum (VD) and convection drying at a temperature of 50 °C (CD50), 60 °C (CD60) and 70 °C (CD70).

The value of the L* parameter ranged from 63.56 to 91.90 for celery without pretreatment, for celery after vacuum impregnation from 37.02 to 58.59. A decrease in the studied parameter was observed in samples after the vacuum impregnation process. This is due to the addition of beet juice, which has a characteristic dark color. Similar observations were noted by other authors who studied apples subjected to osmotic dehydration with chokeberry juice [28] and zucchini and broccoli subjected to vacuum impregnation with beet juice [2]. During the process of vacuum impregnation, atmospheric pressure is lowered, and therefore, the capillaries expand and deform. By lowering the pressure, the capillaries are partially filled with an impregnating solution. In the next stage, VI, the pressure returns to the atmospheric pressure level, and there is a narrowing of the vessels and an intensive flow of the impregnating solution into their interior [13]. For both celery without pretreatment and celery after VI after freeze drying, the highest values of the L* parameter were recorded, demonstrating the brightening of the tested materials in the case of this drying method. These results are in accordance with the studies of other authors [29]. During FD, the temperature of dried vegetables is lowered below the freezing point. Therefore, water is removed from vegetables by sublimation of ice to water vapor, which allows a direct transition from a solid state to a gaseous state, and the liquid phase is skipped. During sublimation drying, the pressure is lower than the triple point of water. Sublimation drying, combining low temperatures and pressure, is considered the best dehydration method, thanks to which it is possible to maintain a very good color, shape and taste of dried products [30,31]. Dries obtained by using the vacuum method for both tested materials were characterized by obtaining the lowest values of the L* parameter (C VD—63.56; CB VD—37.02), as can be seen in Figure 6. Other researchers drying celery at 60 °C observed higher L* values for vacuum-dried celery than convection-dried celery. Differences in color range may probably be due to prolonged drying time by the VD method [29]. Much literature confirms that increasing the drying time causes a decrease in the brightness of vegetables [29,31].

The use of the vacuum impregnation process resulted in a significant increase in the color index a*, which is related to the red shade, as well as an increase in the browning value (BI), saturation (C*) and the color difference ∆E. This is caused by the use of beetroot juice, which is characterized by an intense dark burgundy color. These results are consistent with the observations of other authors who examined the effect of vacuum impregnation using beetroot juice on the properties of zucchini and broccoli [2].

### 2.5. Pearson Correlation of Drying Kinetics and Selected Celery Quality Attributes

**Figure 7:**
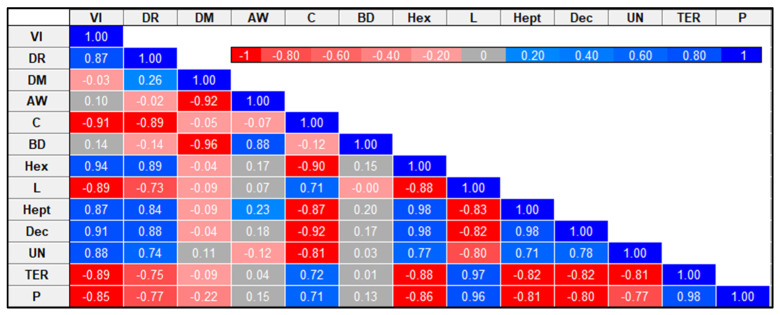
Correlation matrix for selected celery parameters. VI: vacuum impregnation; DR: drying methods; DM: dry matter; AW: water activity; C: color; BD: bulk density; Hex: 1-Hexanol; L: Limonene; Hept: 1-Heptanol; Dec: Decane; UN: unknown sesquiterpene or sesquiterpenoid; TER: γ-Terpinene; P: β-Pinene.

As shown in Figure 7, Pearson’s correlation was used to examine the association between selected celery properties. The data showed a significant (*p* < 0.05) positive correlation between vacuum impregnation and drying methods (r = 0.87), 1-Hexanol (r = 0.94), 1-Heptanol (r = 0.87), Decane (r = 0.91), unknown sesquiterpene or sesquiterpenoid (r = 0.88) and a negative correlation with color (r = −0.91), Limonene (r = −0.89), γ-Terpinene (r = −0.89) and β-Pinene (r = −0.85). The results also showed a significant negative correlation of drying methods with color (r = −0.89) and a significantly positive correlation with 1-Hexanol (r = 0.89), 1-Heptanol (r = 0.84) and Decane (r = 0.88). The data obtained indicate that vacuum impregnation had a greater impact on VOCs than drying methods. Moreover, a significant negative correlation was observed between dry matter and water activity (r = −0.92) and bulk density (r = −96). Water activity positively correlates with density (r = 0.88). The results also showed a positive correlation of color with Limonene (r = 0.72), γ-Terpinene (r = 0.71) and β-Pinene (r = 0.72) and a significant negative correlation with 1-Hexanol (r = −0.90), 1-Heptanol (r = −0.87) and Decane (r = −0.92).

### 2.6. Principal Component Analysis (PCA)

**Figure 8:**
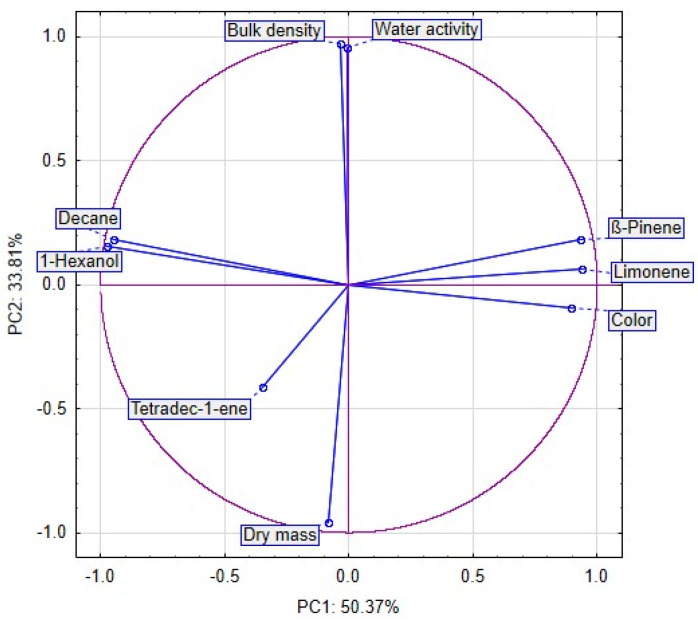
PCA analysis of dried selery.

Approximately 84% of the sample data variance was explained by the first two principal components: PC1 (50.37%) and PC2 (33.81%). PC1 was negatively correlated with the bulk density, water activity, dry mass, Decane, 1-Hexanol and Tetradec-1-ene, and positively with β-Pinnene, Limonene and color of celery. PC2 was negatively correlated with Tetradec-1-ene, color and dry mass and positively correlated with bulk density, water activity, Decane, 1-Hexanol, β-Pinnene and Limonene of celery (Figure 8).

**Figure 9:**
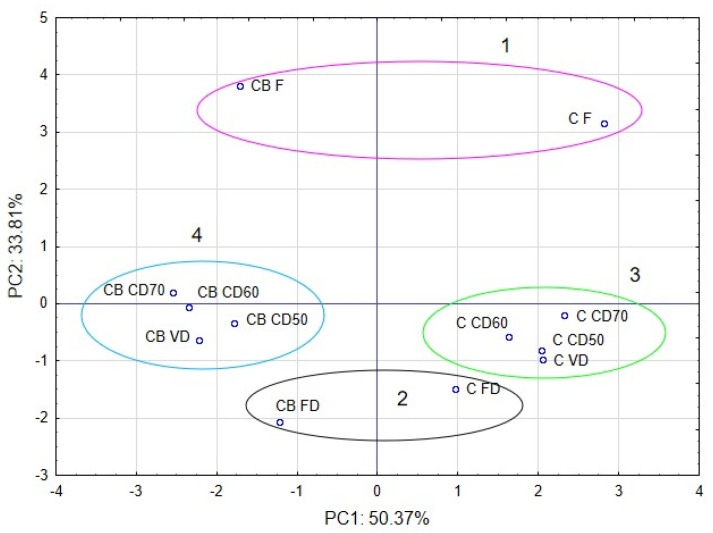
The celery objects in space of first two major components by PCA (principal component analysis).

The PCA plots reveal that the first principal component (PC1) refers to the use of vacuum impregnation process at 50.37%. In the figure, positive values of PC1 describe the results obtained for celery without vacuum impregnation process (Figure 9). The negative values of PC1 describe the celery with vacuum impregnation process. The second principal component (PC2) refers to the use of drying method at 33.81%. In the figure, negative values of PC2 describe the celery after freeze drying. The positive values of PC2 describe the results obtained for fresh celery without drying. Values from 1 to −1 of PC2 describe celery after convection and vacuum drying. Placing the samples in the space of the first two factors showed that the samples were different from each other (Figure 8). Moreover, significant differences can be observed between clusters. The first cluster included sample C F and CB F (fresh celery). The second cluster included sample celery after freeze drying. The next cluster included samples celery without VI and after vacuum and convection drying (3), and the last cluster included samples after VI and vacuum and convection drying (4).

## 3. Materials and Methods

### 3.1. Plant Materials

Celery was purchased from a local vegetable market (Wrocław, Poland) in 2023. The raw material was stored at 4 ± 2 °C in a RL58GRGIH refrigerator (Samsung Electronics Polska sp. z o.o., Wronki, Poland) until testing. Celery with an initial moisture content of 90.11% was washed and peeled, then sliced into 6.5 mm thick slices. Samples for testing were prepared in the shape of wedges measuring 5 × 5 × 3 cm. All recipes are presented in Table 6.

Beet juice (Multi-Smak Sp. z o.o., Warsaw, Poland)—7.9 °Brix—was used as an impregnating solution. The nutritional value of beet juice per 100 mL was as follows: energy value 176 kJ/42 kcal, fat 0.1 g including saturated fatty acids 0.0 g, carbohydrates 7.3 g including sugars 6.9 g, fiber 2.2 g, protein 1.8 g, salt 0.1 g, vitamin C 10 mg/12.5% RWS*.

### 3.2. Vacuum Impregnation (VI)

**Figure 10:**
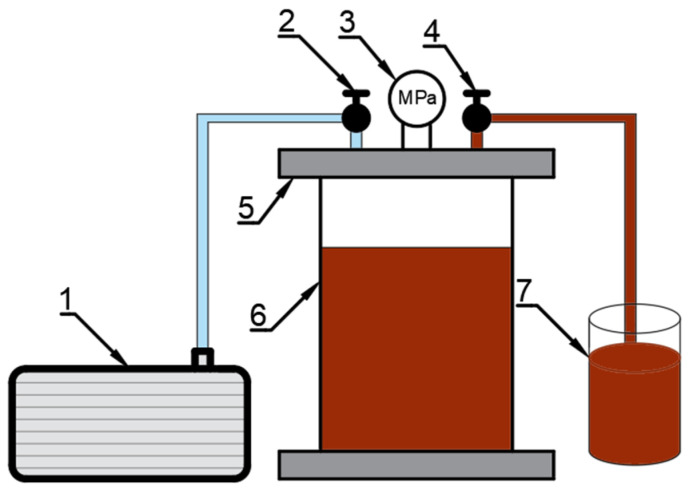
Schematic of the vacuum impregnation apparatus. 1—vacuum pump, 2,4—valves, 3—vacuum gauge, 5—lid, 6—vacuum chamber, 7—impregnation liquid.

Vacuum impregnation of celery was performed in a prototype (Figure 10) device located in the laboratory of the Wrocław University of Life Sciences [32]. The prepared celery weighing 125 g was placed in a perforated vessel made of stainless steel. The vacuum phase of the impregnation process was carried out in a vacuum chamber connected to a vacuum pump at a pressure of 0.06 MPa for 2 min. Then 700 mL of the impregnation solution was added while maintaining a constant pressure of 0.06 MPa for 4 min. During this part of the process, the samples were completely immersed in the impregnating solution, allowing effective saturation of the material. The celery was then left in the impregnating solution for 15 min under atmospheric pressure. After VI, the material was drained using filter paper.

### 3.3. Drying Methods

After the vacuum impregnation process, the material was subjected to drying by using three methods: the freeze drying method (FD), the vacuum method (VD) and the convection method (CD).

#### 3.3.1. Freeze Drying (FD)

After the vacuum impregnation process, the material was immediately frozen at −20 °C for 24 h. The freezing rate was 1 °C·min^−1^ in the RL58GRGIH freezer (Samsung Electronics Polska sp. z o.o., Wronki, Poland). FD was performed in the device (Free-Zone 4.5 L, Labconco, Fort Scott, KS, USA). The drying process lasted 24 h at 5 Pa pressure at −50 °C. The heating shelves reached a temperature of 22 °C.

#### 3.3.2. Vacuum Drying (VD)

Vacuum drying was carried out in a V0101 dryer (Memmert, Schwabach, Germany) at 60 °C under a pressure of 10 kPa. The drying time was 24 h.

#### 3.3.3. Convection Drying (CD)

Convection drying was performed in a dryer designed and built at the Institute of Agricultural Engineering (Wroclaw, Poland) [33]. Samples weighing 40 g were placed in a single layer in baskets. The air flow velocity was 0.5 M·s^−1^. Drying was performed at three temperatures: 50 °C (CD50), 60 °C (CD60) and 70 °C (CD70).

### 3.4. Mathematical Modeling

The moisture ratio was calculated using Equation (1) [18]:

(1) $$MR=\frac{M_{t}-M_{e}}{M_{0}-M_{e}}$$

where: M_t_—water content after time t (g water/g dry weight), M_e_—equilibrium water content (g water/g dry weight), M_0_—initial water content (g water/g dry weight)

In order to select the best mathematical model to describe the convection drying of celery root, five equations commonly cited in the literature were analyzed (Table 7).

### 3.5. Volatile Organic Compounds (VOCs)

#### Headspace Solid-Phase Microextraction (HS-SPME Arrow)—GC-MS

Volatile organic compound (VOC) analysis was performed with HS-SPME technique. Amounts of 0.2 ± 0.005 g of dried and 2 ± 0.010 g of fresh material (previously homogenized) were weighed in sequence and put into 20 mL headspace glass vials. Then, 2-undecanone (Sigma-Aldrich, Steinheim, Germany) was added as an internal standard in the amount of 5 µL (1 mg/mL). HS-SPME extraction was performed using a 1.10 mm DVB/C-WR/PDMS SPME Arrow fiber (Shimadzu, Kyoto, Japan). Before each analysis, the fiber was pre-conditioned at 250 °C for 5 min. Extraction was preceded by sample incubation for 5 min in 60 °C, and thereafter the VOC extraction was carried out for 30 min at 60 °C. Desorption of the sample lasted 3 min in the GC injection port. Analyte separation and identification were performed on a Shimadzu QP 2020 Plus (Shimadzu, Kyoto, Japan) equipped with a ZB-5Msi column (Phenomenex, Torrance, CA, USA) (30 m × 0.25 mm × 0.25 µm). The injector temperature was 250 °C, and the carrier gas was helium at a flow rate of 1.0 mL·min^−1^ with a linear velocity of 36.3 cm·s^−1^ and a split of 20. The temperature program was 50 °C, then 130 °C at a rate of 4 °C min^−1^; then 180 °C at a rate of 10 °C min^−1^; then 280 °C at a rate of 20 °C min^−1^. The ion source temperature and interface temperature were 220 and 250 °C, respectively. The MS mode was scan 40–400 *m*/*z*. The analyses were carried out in triplicate.

A comparison of the obtained mass spectra (at least 90%) and a comparison of the calculated linear retention indices (LRI) (±15) with the NIST 20 (National Institute of Standards and Technology, Gaithersburg, MD, USA) and FFNSC (Mass Spectra of Flavors and Fragrances of Natural and Synthetic Compounds) databases were used to identify the analyses. The quantification was obtained by area normalization method against the internal standard peak.

### 3.6. Physical Properties

#### 3.6.1. Dry Weight, Water Activity, Bulk Density

Dry weight of celery samples was determined gravimetrically by drying at 70 °C for 24 h under reduced pressure (5 kPa) in a vacuum dryer (Memmert, VO101, Schwabach, Germany). The measurement was performed in triplicate, and the average of the measurements was taken as the result.

The water activity of the tested samples was determined using AquaLab 4TE ± 0.003 (AquaLab, Warsaw, Poland). Measurements were made at a constant temperature of 25 °C. The result obtained was the average of four measurements.

Bulk density was taken using a measuring cylinder, which was filled with the material [39]. The measurement was made in six repetitions and calculated according to the formula:

(2) $$\rho_{b}=\frac{w_{s}}{V}$$

where: ρ_b_—bulk density [kg · m^−3^], w_s_—weight of samples [kg], V—volume [m^3^].

#### 3.6.2. Color

Using a colorimeter (Minolta Chroma Meter CR-200 colorimeter (Minolta Corp., Osaka, Japan)), the color indices L* (brightness), a* (red-green) and b* (yellow-blue) were obtained. And also, the overall color change (ΔE), the browning index (BI) and color saturation (C*) were calculated using the following equations [40]

Total color change (ΔE) was calculated according to equation:

(3) $$∆E=\sqrt{\left[{\left(L_{sample}^{*}-L_{control}^{*}\right)}^{2}+{\left(a_{sample}^{*}-a_{control}^{*}\right)}^{2}+{\left(b_{sample}^{*}-b_{control}^{*}\right)}^{2}\right]}$$

where:

L*_control_, a*_control_, and b*_control_—color of fresh celery

L*_sample_, a*_sample_, and b*_sample_—color of dried celery samples

The color saturation (C*):

(4) $$C*=\sqrt{{a*}^{2}+{b*}^{2}}$$

The browning Index (BI):

(5) $$BI=100\cdot \left(\frac{X-0.31}{0.17}\right)$$

where:

(6) $$X=\frac{a*+1.75L*}{5.645L*+a*-3.012b*}$$

### 3.7. Statistical Analysis

Statistical analyses were performed using Statistica version 13.1 (StatSoft, Tulsa, OK, USA). One-way analysis of variance (ANOVA) using Duncan’s test was used to compare the mean values. Differences were considered to be significant at *p* < 0.05. The significant differences in major VOCs (abundance in fresh samples at least 1%) were verified by hierarchical cluster analysis (HCA). Numerical data used before the analysis were standardized according to software algorithm. Assumptions for HCA included Ward’s linkage, Euclidean distance and the strict (33%) Sneath’s criterium. Principal component analysis (PCA) was also performed for the tested selected physical properties and volatile compounds of celery depending on the drying method and vacuum impregnation applied. A dendrogram plot was then prepared to analyze the clustered data.

## 4. Conclusions

In this study, we investigated the effect of pretreatment, such as vacuum impregnation, and various drying process methods and parameters on the drying properties and selected quality characteristics and VOCs of dried celery. The results showed that pretreatment significantly reduced the drying time of celery and increased their drying rate, regardless of the temperature of the drying medium used. In addition, the dries that were obtained using VI were characterized by lower dry matter and increased water activity and density. The use of beet juice as an impregnating solution significantly reduced the brightness and increased the shade of red in the samples tested. Moreover, the vacuum impregnation process influenced the content of volatile compounds, which is related to the addition of beetroot juice. The amounts of major compounds such as limonene (36.02%), pentyl cyclohexa-1,3-diene (3.22%) and Terpinene <gamma-> (3.71%) decreased after impregnation. The effect of different drying conditions on the quality of the dried samples was also studied. Drying methods had a significant effect on water activity, density, color, drying time and VOCs. Increasing the temperature of the drying medium during convection drying reduced the drying time and increased the values of dry weight and water activity. Of all the mathematical models tested, the logistic model had the best fit. Droughts obtained by using the sublimation method had the lowest water activity values and the lowest bulk density. In addition, the study confirmed that dries obtained by using the sublimation method retained their color and shape better than those obtained by using other methods.

## 5. Patents

Patent Poland, no. 421913. Vacuum impregnating machine and method for initial processing of material. Wrocław University of Environmental and Life Sciences, Wrocław, PL. Authors: Bogdan Stępień, Radosław Maślankowski, Leszek Rydzak, and Marta Pasławska.

## Figures and Tables

**Table 1:** HS-SPME Arrow VOCs profile for fresh celery. Celery (C) after freeze drying (FD), vacuum (VD) and convection drying at a temperature of 50 °C (CD50), 60 °C (CD60) and 70 °C (CD70).

Compounds	LRI Exp ^1^	LRI Lit ^2^	Match ^3^	C	C VD	C CD50	C CD60	C CD70	C FD
Hexanal	800	806	97	0.44 ± 0.13	0.21 ± 0.01	0.48 ± 0.12	0.15 ± 0.00	0.15 ± 0.02	0.28 ± 0.01
1-Hexanol	864	860	91	0.02 ± 0.02	0.41 ± 0.02	0.36 ± 0.04	0.37 ± 0.08	0.25 ± 0.03	0.95 ± 0.05
Nonane	899	900	97	2.99 ± 0.32	3.62 ± 0.31	4.00 ± 0.32	3.58 ± 1.09	4.94 ± 0.06	1.37 ± 0.41
Butyrolactone	912	941	92	0.00 ± 0.00	0.39 ± 0.02	0.37 ± 0.01	0.04 ± 0.01	0.44 ± 0.10	0.03 ± 0.02
α-Pinene	933	933	94	0.44 ± 0.06	0.37 ± 0.02	0.25 ± 0.00	0.21 ± 0.03	0.43 ± 0.01	0.13 ± 0.01
1-Heptanol	967	970	90	0.03 ± 0.00	0.12 ± 0.02	0.05 ± 0.01	0.10 ± 0.01	0.05 ± 0.01	0.93 ± 0.05
β-Pinene	976	978	99	11.78 ± 2.12	9.94 ± 0.57	5.87 ± 0.09	5.51 ± 0.68	9.45 ± 0.18	2.39 ± 0.35
Myrcene	990	991	98	3.77 ± 0.49	3.44 ± 0.10	2.68 ± 0.16	2.37 ± 0.01	5.37 ± 0.58	0.92 ± 0.13
Decane	999	1000	95	0.13 ± 0.03	0.94 ± 0.02	0.43 ± 0.04	0.39 ± 0.18	0.55 ± 0.14	2.75 ± 1.46
Octanal	1002	1005	95	1.80 ± 0.21	0.16 ± 0.01	0.25 ± 0.03	0.13 ± 0.01	0.18 ± 0.03	0.33 ± 0.06
p-Cymen	1024	1025	98	5.55 ± 1.32	2.03 ± 0.09	1.44 ± 0.03	1.53 ± 0.16	1.40 ± 0.13	1.05 ± 0.11
Limonene	1028	1030	98	43.91 ± 3.09	32.56 ± 0.60	32.91 ± 0.45	27.35 ± 0.01	46.38 ± 0.49	9.80 ± 1.49
β-(E)-Ocimene	1036	1046	95	6.61 ± 1.01	9.02 ± 0.40	5.62 ± 0.02	6.23 ± 0.40	9.36 ± 0.58	2.26 ± 0.38
γ-Terpinene	1058	1058	97	3.64 ± 0.66	4.09 ± 0.24	2.48 ± 0.12	2.43 ± 0.28	3.69 ± 0.23	0.96 ± 0.14
1-Octanol	1069	1076	91	0.13 ± 0.03	0.19 ± 0.03	0.07 ± 0.02	0.16 ± 0.04	0.04 ± 0.01	0.76 ± 0.07
Undecane	1098	1100	94	0.23 ± 0.12	0.50 ± 0.12	0.28 ± 0.01	0.40 ± 0.03	0.26 ± 0.03	0.63 ± 0.11
Pentyl cyclohexa-1,3-diene	1157	1160	92	5.72 ± 0.86	8.69 ± 0.02	19.64 ± 1.17	28.51 ± 1.08	6.47 ± 0.44	2.38 ± 0.10
α-Thujene	1198	1200	96	0.48 ± 0.04	0.52 ± 0.26	1.14 ± 0.01	1.26 ± 0.11	0.37 ± 0.05	0.21 ± 0.03
Dodecane	1262		97	0.29 ± 0.03	8.08 ± 0.06	3.23 ± 0.54	2.78 ± 0.03	3.41 ± 0.18	31.98 ± 1.51
2-Methyldodecane	1262	1249	92	0.01 ± 0.00	0.34 ± 0.01	0.17 ± 0.03	0.13 ± 0.01	0.14 ± 0.04	1.52 ± 0.12
Tridecane	1298	1300	94	0.45 ± 0.57	0.29 ± 0.00	0.18 ± 0.03	0.14 ± 0.03	0.15 ± 0.03	0.98 ± 0.09
Tetradec-1-ene	1392	1392	95	0.03 ± 0.00	0.14 ± 0.38	0.22 ± 0.01	0.15 ± 0.03	0.09 ± 0.03	0.24 ± 0.02
Tetradecane	1399	1400	96	0.27 ± 0.02	4.07 ± 0.02	2.80 ± 0.55	1.79 ± 0.26	2.49 ± 0.53	15.28 ± 1.62
(*E*)-Caryophyllene	1426	1424	96	0.12 ± 0.00	0.60 ± 0.02	0.90 ± 0.06	0.38 ± 0.13	0.43 ± 0.18	0.12 ± 0.02
α-trans-Bergamotene	1441	1432	96	0.07 ± 0.02	0.29 ± 0.00	0.45 ± 0.01	0.75 ± 0.02	0.11 ± 0.03	0.06 ± 0.01
β-(*E*)-Farnesene + Humulene	1461	1452/1454	91/90	0.02 ± 0.00	0.13 ± 0.02	0.22 ± 0.03	0.12 ± 0.05	0.10 ± 0.04	0.03 ± 0.01
β-Selinene	1493	1492	94	0.15 ± 0.03	0.30 ± 0.02	0.66 ± 0.03	0.24 ± 0.09	0.35 ± 0.12	0.07 ± 0.00
α-Selinene	1501	1501	90	0.02 ± 0.00	0.03 ± 0.00	0.10 ± 0.01	0.04 ± 0.02	0.05 ± 0.02	0.02 ± 0.00
unknown sesquiterpene or sesquiterpenoid	1522			0.05 ± 0.01	0.04 ± 0.00	0.04 ± 0.01	0.03 ± 0.01	0.03 ± 0.01	0.02 ± 0.00
Hexadecane	1599	1600	97	0.11 ± 0.02	0.49 ± 0.06	0.37 ± 0.08	0.24 ± 0.03	0.39 ± 0.10	1.99 ± 0.22
unknown sesquiterpene or sesquiterpenoid	1602			0.06 ± 0.01	0.07 ± 0.01	0.04 ± 0.01	0.05 ± 0.02	0.03 ± 0.01	0.15 ± 0.02
unknown sesquiterpene or sesquiterpenoid	1607			0.06 ± 0.01	0.03 ± 0.00	0.13 ± 0.02	0.12 ± 0.01	0.01 ± 0.00	0.06 ± 0.01
3-Butyl hexahydro phthalide	1647	1631	90	0.33 ± 0.09	0.13 ± 0.00	0.66 ± 0.05	0.35 ± 0.04	0.09 ± 0.03	0.22 ± 0.02
3-Butyl phthalide	1650	1648	90	2.63 ± 0.61	1.06 ± 0.10	1.93 ± 0.07	1.70 ± 0.16	0.60 ± 0.01	2.59 ± 0.33
(3*Z*)-Butylidene phthalide	1683	1673	90	0.34 ± 0.07	0.17 ± 0.03	0.20 ± 0.02	0.14 ± 0.02	0.08 ± 0.00	0.36 ± 0.04
Fenipentol	1740	nd ^4^	tr ^5^	1.63 ± 0.20	2.57 ± 0.38	1.92 ± 0.12	1.23 ± 0.19	0.82 ± 0.00	7.28 ± 1.07
3-Isobutylidene phthalide	1746	1722	97	2.30 ± 0.21	2.26 ± 0.27	5.85 ± 0.63	4.86 ± 0.02	0.47 ± 0.08	4.98 ± 0.67
(*Z*)-Ligustilide	1748	1733	94	0.24 ± 0.05	0.45 ± 0.07	0.20 ± 0.02	0.21 ± 0.06	0.10 ± 0.01	0.85 ± 0.11
Neocnidilide	1750	1735	94	3.17 ± 0.67	1.25 ± 0.05	1.87 ± 0.33	4.03 ± 0.14	0.37 ± 0.06	3.08 ± 0.38

^1^ LRI exp—experimentally calculated LRI; ^2^ LRI lit—LRI available in library; ^3^ Mass spectra similiraty match [%], ^4^ nd—not detected; ^5^ tr—trace (<0.05%).

**Table 2:** HS-SPME Arrow VOCs profile for fresh celery impregnated with beetroot juice. Celery after vacuum impregnation with beetroot juice (CB) after freeze drying (FD), vacuum (VD) and convection drying at a temperature of 50 °C (CD50), 60 °C (CD60) and 70 °C (CD70).

Compounds	LRI Exp ^1^	LRI Lit ^2^	Match ^3^	CB	CB VD	CB CD50	CB CD60	CB CD70	CB FD
Hexanal	802	806	97	0.41 ± 0.04	1.25 ± 0.26	0.56 ± 0.18	0.18 ± 0.06	0.15 ± 0.02	0.98 ± 0.09
Nonane	901	900	96	4.17 ± 0.28	3.81 ± 0.60	3.65 ± 0.15	4.54 ± 1.62	5.16 ± 0.06	2.37 ± 0.64
α-Pinene	934	933	97	0.56 ± 0.04	0.39 ± 0.02	0.34 ± 0.02	0.37 ± 0.00	0.45 ± 0.01	0.22 ± 0.02
Sabinene	972	972	97	0.27 ± 0.02	0.12 ± 0.01	0.15 ± 0.02	0.18 ± 0.00	0.15 ± 0.00	0.09 ± 0.01
β-Pinene	977	978	98	15.86 ± 2.07	8.24 ± 0.26	8.52 ± 0.81	8.48 ± 0.16	9.86 ± 0.15	3.60 ± 0.66
Myrcene	991	991	97	4.18 ± 0.54	3.88 ± 0.26	3.16 ± 0.33	4.34 ± 0.22	5.61 ± 0.62	1.21 ± 0.26
Octanal	1003	1005	96	1.74 ± 0.08	0.44 ± 0.07	1.27 ± 0.28	0.34 ± 0.10	0.19 ± 0.03	0.71 ± 0.02
p-Cymene	1024	1025	98	6.60 ± 1.60	1.80 ± 0.10	3.07 ± 0.42	2.23 ± 0.05	1.46 ± 0.13	2.06 ± 0.05
Limonene	1030	1030	96	36.02 ± 4.47	42.34 ± 1.36	35.07 ± 1.73	45.31 ± 2.23	48.41 ± 0.65	13.16 ± 1.98
β-(*E*)-Ocimene	1037	1045	95	7.47 ± 0.72	6.66 ± 0.20	5.84 ± 0.27	8.25 ± 1.06	9.77 ± 0.63	2.82 ± 0.50
γ-Terpinene	1058	1058	97	3.71 ± 0.55	3.31 ± 0.31	2.85 ± 0.20	3.70 ± 0.49	3.85 ± 0.23	1.45 ± 0.22
(5*Z*)-Octen-1-ol	1076	1073	tr ^4^	0.25 ± 0.04	0.02 ± 0.00	0.01 ± 0.00	0.01 ± 0.00	0.01 ± 0.00	0.06 ± 0.04
Nonanal	1103	1104	95	0.22 ± 0.03	0.27 ± 0.06	0.65 ± 0.26	0.24 ± 0.07	0.16 ± 0.03	0.63 ± 0.17
cis-Limonene oxide	1133	1134	97	0.34 ± 0.06	0.03 ± 0.00	0.14 ± 0.12	0.09 ± 0.08	0.01 ± 0.01	0.03 ± 0.02
trans-Limonene oxide	1137	1138	90	2.30 ± 0.40	0.05 ± 0.01	0.09 ± 0.01	0.05 ± 0.00	0.04 ± 0.00	0.12 ± 0.08
Pentyl cyclohexa-1,3-diene	1158	1160	90	3.22 ± 0.79	7.32 ± 1.26	3.19 ± 0.11	5.77 ± 1.81	6.75 ± 0.47	3.81 ± 0.09
Decanal	1204	1204	92	0.18 ± 0.03	2.31 ± 0.47	0.18 ± 0.15	0.00 ± 0.00	0.42 ± 0.42	12.57 ± 0.22
trans-Carveol	1219	1223	90	0.15 ± 0.03	0.00 ± 0.00	1.07 ± 0.30	0.01 ± 0.01	0.00 ± 0.00	0.01 ± 0.01
(2*E*)-Decenal	1261	1265	96	0.15 ± 0.03	0.03 ± 0.02	0.07 ± 0.02	0.02 ± 0.01	0.03 ± 0.01	0.23 ± 0.22
Triacetin	1356	1354	91	0.19 ± 0.09	0.36 ± 0.13	2.05 ± 1.84	0.50 ± 0.40	0.06 ± 0.03	0.69 ± 0.05
Tetradecene	1392	1392	92	0.03 ± 0.00	0.19 ± 0.01	0.11 ± 0.04	0.15 ± 0.02	0.09 ± 0.03	0.39 ± 0.02
Tetradecane	1399	1400	90	0.03 ± 0.00	6.25 ± 1.09	1.34 ± 0.21	3.25 ± 0.89	2.60 ± 0.55	26.73 ± 1.75
Methyl eugenol	1406	1403	85	0.08 ± 0.05	0.01 ± 0.00	0.08 ± 0.02	0.05 ± 0.01	0.02 ± 0.01	0.00 ± 0.00
Dodecanal	1409	1402	94	0.05 ± 0.01	0.02 ± 0.01	0.05 ± 0.02	0.01 ± 0.01	0.01 ± 0.00	0.16 ± 0.01
(*E*)-Caryophyllene	1426	1424	96	0.11 ± 0.02	0.78 ± 0.11	0.41 ± 0.29	0.51 ± 0.02	0.45 ± 0.19	0.20 ± 0.02
α-trans-Bergamotene	1441	1432	96	0.09 ± 0.02	0.19 ± 0.02	0.22 ± 0.10	0.22 ± 0.05	0.12 ± 0.03	0.10 ± 0.02
trans-Geranylacetone	1456	1450	92	0.03 ± 0.01	0.03 ± 0.00	0.07 ± 0.02	0.04 ± 0.01	0.02 ± 0.01	0.12 ± 0.02
β-(*E*)-Farnesene	1461	1452	90	0.02 ± 0.00	0.06 ± 0.00	0.03 ± 0.01	0.06 ± 0.01	0.04 ± 0.02	0.04 ± 0.00
α-Humulene	1462	1454	90	0.02 ± 0.01	0.17 ± 0.02	0.15 ± 0.12	0.15 ± 0.00	0.10 ± 0.04	0.05 ± 0.01
unknown sesquiterpene or sesquiterpenoid	1469			0.17 ± 0.05	0.96 ± 0.17	1.87 ± 0.46	0.99 ± 0.27	0.58 ± 0.18	2.90 ± 0.08
Dodecanol	1477	1476	tr	0.04 ± 0.02	0.01 ± 0.00	0.04 ± 0.01	0.02 ± 0.01	0.01 ± 0.00	0.02 ± 0.01
unknown sesquiterpene or sesquiterpenoid	1482			0.43 ± 0.13	1.05 ± 0.26	0.76 ± 0.91	0.42 ± 0.34	0.49 ± 0.46	0.62 ± 0.03
β-Selinene	1494	1492	94	0.14 ± 0.03	0.57 ± 0.04	0.68 ± 0.60	0.60 ± 0.13	0.36 ± 0.12	0.10 ± 0.01
α-Selinene	1502	1501	86	0.02 ± 0.00	0.09 ± 0.00	0.10 ± 0.10	0.10 ± 0.02	0.05 ± 0.02	0.01 ± 0.00
unknown sesquiterpene or sesquiterpenoid	1523			0.04 ± 0.01	0.04 ± 0.00	0.06 ± 0.03	0.04 ± 0.00	0.03 ± 0.01	0.03 ± 0.00
Caryophyllene oxide	1596	1587	90	0.02 ± 0.00	0.01 ± 0.00	0.01 ± 0.00	0.01 ± 0.00	0.00 ± 0.00	0.02 ± 0.01
Tetradecanal	1615	1601	90	0.01 ± 0.00	0.02 ± 0.00	0.25 ± 0.32	0.01 ± 0.00	0.01 ± 0.00	0.04 ± 0.01
Methyl trans-dihydrojasmonate	1663	1648	94	0.11 ± 0.04	0.05 ± 0.00	0.24 ± 0.08	0.06 ± 0.03	0.04 ± 0.00	0.20 ± 0.05
3-Butyl phthalide	1664	1648	91	1.72 ± 0.59	0.89 ± 0.59	3.31 ± 0.22	1.61 ± 0.54	0.32 ± 0.32	0.01 ± 0.00
cis-9-Tetradecen-1-ol	1677	1664	T	0.23 ± 0.03	0.21 ± 0.04	0.50 ± 0.10	0.19 ± 0.04	0.10 ± 0.01	0.52 ± 0.08
(3*Z*)-Butylidene phthalide	1689	1673	92	0.39 ± 0.06	0.18 ± 0.03	0.43 ± 0.04	0.12 ± 0.04	0.08 ± 0.00	0.48 ± 0.06
Fenipentol	1740	nd ^5^	tr	2.74 ± 0.56	2.49 ± 0.30	6.14 ± 1.75	2.41 ± 0.69	0.85 ± 0.01	8.64 ± 0.62
3-Isobutylidene phthalide	1746	1722	97	2.40 ± 0.14	1.85 ± 0.17	5.07 ± 0.61	2.28 ± 0.16	0.49 ± 0.09	5.67 ± 0.01
(*Z*)-Ligustilide	1748	1733	94	0.57 ± 0.14	0.17 ± 0.01	0.56 ± 0.09	0.27 ± 0.09	0.11 ± 0.02	0.95 ± 0.10
Neocnidilide	1751	1735	94	2.07 ± 0.80	1.00 ± 0.22	3.88 ± 0.62	1.39 ± 0.03	0.38 ± 0.06	4.59 ± 029
Octyl caprylate	1780	1779	91	0.06 ± 0.02	0.01 ± 0.00	0.06 ± 0.03	0.05 ± 0.01	0.01 ± 0.01	0.16 ± 0.14
Octadecane	1799	1800	90	0.06 ± 0.01	0.05 ± 0.01	0.13 ± 0.03	0.05 ± 0.01	0.03 ± 0.01	0.29 ± 0.02
Isopropyl tetradecanoate	1826	1826	93	0.25 ± 0.08	0.03 ± 0.01	0.20 ± 0.02	0.05 ± 0.01	0.04 ± 0.01	0.15 ± 0.03
Methyl 14-methylhexadecanoate	1928	1914	T	0.04 ± 0.02	0.01 ± 0.00	1.28 ± 0.31	0.41 ± 0.07	0.10 ± 0.01	0.10 ± 0.01
Isopropyl palmitate	2025	2013	94	0.04 ± 0.02	0.01 ± 0.00	0.03 ± 0.01	0.01 ± 0.01	0.01 ± 0.01	0.02 ± 0.00

^1^ LRI exp– experimentally calculated LRI; ^2^ LRI lit—LRI available in library; ^3^ Mass spectra similarity match [%], ^4^ tr—trace ^5^ nd—not detected; (<0.05%).

**Table 3:** Model parameters and statistics describing the drying kinetics of celery dried by convection at different temperatures. Celery (C), celery after vacuum impregnation with beetroot juice (CB), material after freeze drying (FD), vacuum (VD) and convection drying at a temperature of 50 °C (CD50), 60 °C (CD60) and 70 °C (CD70).

Drying Method	Material	Model Parameters			Statistical Parameters				Drying Time [min]
		k	a	b	RMSE	V_e_ [%]	R^2^	χ^2^	
Logistic Model									
CD50	CB	0.0134	5.4908	6.4022	0.0099	0.0230	0.9992	0.0001	330
	C	0.0130	1.5099	2.4754	0.0108	0.0235	0.9991	0.0001	400
CD60	CB	0.0206	2.4017	3.3431	0.0109	0.0264	0.9989	0.0001	240
	C	0.0171	2.3372	3.2945	0.0084	0.0199	0.9994	0.0001	300
CD70	CB	0.0236	2.9346	3.8320	0.0132	0.0338	0.9984	0.0002	210
	C	0.0209	1.0870	2.0494	0.0107	0.0236	0.9990	0.0001	240
Logarithmic Model									
CD50	CB	0.0111	1.0381	-	0.0157	0.0370	0.9978	0.0003	330
	C	0.0089	1.0546	-	0.0208	0.0451	0.9968	0.0005	400
CD60	CB	0.0155	1.0708	-	0.0173	0.0419	0.9974	0.0003	240
	C	0.0125	1.0862	-	0.0311	0.0722	0.9920	0.001	300
CD70	CB	0.0190	1.0827	-	0.0191	0.0493	0.9967	0.0004	210
	C	0.0138	1.0790	-	0.0235	0.0529	0.9956	0.0006	240
Henderdon and Pabis Model									
CD50	CB	0.0125	0.9945	-	0.0108	0.0256	0.9991	0.0001	330
	C	0.0101	1.0195	-	0.0190	0.0412	0.9976	0.0004	400
CD60	CB	0.0174	1.0056	-	0.0154	0.0373	0.9982	0.0003	240
	C	0.0140	1.0325	-	0.0344	0.0800	0.9903	0.0014	300
CD70	CB	0.0211	1.0007	-	0.0156	0.0404	0.9981	0.0003	210
	C	0.0154	1.0215	-	0.0239	0.0538	0.9956	0.0007	240
Newton Model									
CD50	CB	0.0126	-	-	0.0111	0.0261	0.9990	0.0001	330
	C	0.0098	-	-	0.0209	0.0452	0.9983	0.0005	400
CD60	CB	0.0172	-	-	0.0155	0.0377	0.9984	0.0003	240
	C	0.0134	-	-	0.0370	0.0860	0.9912	0.0016	300
CD70	CB	0.0211	-	-	0.0156	0.0404	0.9981	0.0003	210
	C	0.0150	-	-	0.0256	0.0576	0.9966	0.0008	240
Page Model									
CD50	CB	0.0124	1.0037	-	0.0110	0.0261	0.9990	0.0001	330
	C	0.0063	1.0991	-	0.0124	0.0268	0.9988	0.0002	400
CD60	CB	0.0141	1.0496	-	0.0130	0.0316	0.9985	0.0002	240
	C	0.0072	1.1481	-	0.0285	0.0662	0.9929	0.0009	300
CD70	CB	0.0183	1.0368	-	0.0143	0.0371	0.9982	0.0002	210
	C	0.0091	1.1202	-	0.0161	0.0362	0.9978	0.0003	240

**Table 4:** Dry matter (DM), water activity (AW), bulk density (ρb).

Method	DM (%)	AW (-)	ρb (kg·m^–3^)
C	9.89 ± 0.009 ^a^	0.925 ± 0.023 ^i^	221.32 ± 27.30 ^d^
C FD	99.26 ± 0.005 ^f^	0.110 ± 0.010 ^a^	34.28 ± 6.12 ^a^
C VD	98.22 ± 0.019 ^e,f^	0.133 ± 0.036 ^a,b^	58.82 ± 8.71 ^b^
C CD50	89.58 ± 0.012 ^b,c^	0.480 ± 0.018 ^g^	72.04 ± 14.49 ^b^
C CD60	96.44 ± 0.014 ^e,f^	0.363 ± 0.002 ^e^	70.84 ± 13.43 ^b^
C CD70	97.75 ± 0.013 ^e,f^	0.187 ± 0.005 ^c^	67.76 ± 11.73 ^b^
CB	10.43 ± 0.003 ^a^	0.983 ± 0.001 ^j^	271.43 ± 31.34 ^e^
CB FD	98.18 ± 0.009 ^e,f^	0.149 ± 0.011 ^b^	34.48 ± 2.19 ^a^
CB VD	95.51 ± 0.009 ^e,f^	0.180 ± 0.018 ^c^	91.67 ± 19.65 ^c^
CB CD50	87.69 ± 0.025 ^b^	0.557 ± 0.012 ^h^	94.24 ± 10.46 ^c^
CB CD60	91.64 ± 0.182 ^c,d^	0.422 ± 0.005 ^f^	87.79 ± 5.68 ^b^
CB CD70	94.42 ± 0.027 ^d,e^	0.252 ± 0.003 ^d^	63.92 ± 2.26 ^b^

Values (mean of three replications) ± standard deviation followed by different letters (a–j), are different (*p* ≤ 0.05) according to Duncan’s test.

**Table 5:** Color parameters of raw and dried celery. Color parameters of raw and dried celery: L*—lightness, a* for (+) redness/(−) greenness, and b* for (+) yellowing, BI—browning index, C*—saturation, ΔE—total color of vegetables.

Method	L*	a*	b*	BI	C*	∆E
C	85.62 ± 1.3 ^f^	−0.51 ± 0.35 ^a^	10.72± 1.12 ^c^	12.61	10.73	-
C FD	91.90 ± 2.07 ^g^	−0.46 ± 0.15 ^a^	11.38± 0.52 ^c^	12.54	11.39	6.31
C VD	63.56 ± 4.70 ^d^	0.56 ± 0.33 ^a,b^	11.80± 1.33 ^c^	20.71	11.81	22.11
C CD50	77.30 ± 2.55 ^d^	2.50 ± 0.92 ^b^	18.06± 1.54 ^e^	28.44	18.23	11.49
C CD60	75.26 ± 2.85 ^d^	1.61 ± 0.55 ^a,b^	16.60± 1.09 ^d^	25.96	16.68	12.10
C CD70	76.16 ± 1.55 ^d^	2.12 ± 0.83 ^b^	17.67± 1.77 ^d^	27.90	17.80	12.03
CB	43.72 ± 2.35 ^b^	28.81 ± 3.13 ^e^	8.92± 0.76 ^b^	66.75	30.16	51.17
CB FD	58.59 ± 2.14 ^c^	28.06 ± 1.68 ^e^	8.84± 1.96 ^b^	48.90	29.41	39.37
CB VD	37.02 ± 3.66 ^a^	15.64 ± 2.77 ^c^	6.25± 2.52 ^a^	47.54	16.84	51.41
CB CD50	40.26 ± 1.54 ^a,b^	18.22 ± 1.18 ^d^	10.92± 0.94 ^c^	63.00	21.92	49.07
CB CD60	39.65 ± 2.96 ^a,b^	19.37 ± 2.07 ^d^	11.37± 1.30 ^c^	67.52	22.46	50.09
CB CD70	39.59 ± 1.91 ^a,b^	18.90 ± 1.08 ^d^	11.11± 1.62 ^c^	65.93	21.24	49.96

Values (mean of three replications) ± standard deviation followed by different letters (a–g), are different (*p* ≤ 0.05) according to Duncan’s test.

**Table 6:** Samples codes for raw and dried celery.

Code	Material	Type of Drying
C	Celery	-
C FD	Celery	freeze drying
C VD	Celery	vacuum drying
C CD50	Celery	convective drying 50 °C
C CD60	Celery	convective drying 60 °C
C CD70	Celery	convective drying 70 °C
CB	Celery after impregnation with beetroot juice	-
CB FD	Celery after impregnation with beetroot juice	freeze drying
CB VD	Celery after impregnation with beetroot juice	vacuum drying
CB CD50	Celery after impregnation with beetroot juice	convective drying 50 °C
CB CD60	Celery after impregnation with beetroot juice	convective drying 60 °C
CB CD70	Celery after impregnation with beetroot juice	convective drying 70 °C

**Table 7:** Models taken advantage for description of course convective drying.

Number	Model Name	Equation
1	Page Model [34]	$MR=exp(-k\cdot \tau^{a})$
2	Henderdon and Pabis Model [35]	MR=a·exp⁡(−kτ)
3	Newton Model [36]	MR=exp⁡(−kτ)
4	Logarithmic Model [37]	$MR=a\cdot \exp\left(-k\tau \right)+b\cdot exp(-k_{ja}\cdot \tau )$
5	Logistic Model [38]	$MR=\frac{b}{\left(1+a\cdot \exp\left(k\cdot \tau \right)\right)}$

k—drying coefficient [min^−1^]; a, b—coefficients of the equations; n—exponent; τ—time [min]; MR—moisture ratio.

## Data Availability

Data are contained within the article.
